# Endovascular Thrombectomy for Acute Basilar Artery Occlusion: Latest Findings and Critical Thinking on Future Study Design

**DOI:** 10.1007/s12975-022-01008-5

**Published:** 2022-03-29

**Authors:** Wengui Yu, Randall T. Higashida

**Affiliations:** 1 Department of Neurology, University of California, Irvine, 200 S. Manchester Ave., 206E, Orange, CA 92868 USA; 2 Departments of Radiology & Biomedical Imaging, Neurosurgery, Neurology, & Anesthesiology, University of California, San Francisco, CA USA

**Keywords:** Acute ischemic stroke, Basilar artery occlusion, Endovascular thrombectomy, Outcome, Randomized controlled trial

## Abstract

Randomized controlled trials (RCTs) have demonstrated powerful efficacy of endovascular thrombectomy (EVT) for large vessel occlusion in the anterior circulation. The effect of EVT for acute basilar artery occlusion (BAO) in the posterior circulation remains unproven. Here, we highlight the latest findings of observational studies and RCTs of EVT for BAO, with a focus on the predictors of functional outcomes, the limitations of recent RCTs, and critical thinking on future study design. Pooled data from large retrospective studies showed 36.4% favorable outcome at 3 months and 4.6% symptomatic intracranial hemorrhage (sICH). Multivariate logistic regression analysis revealed that higher baseline NIHSS score, pc-ASPECTS < 8, extensive baseline infarction, large pontine infarct, and sICH were independent predictors of poor outcome. Two recent randomized trial BEST (Endovascular treatment vs. standard medical treatment for vertebrobasilar artery occlusion) and BASICS (Basilar Artery International Cooperation Study) failed to demonstrate significant benefit of EVT within 6 or 8 h after stroke symptom onset. The limitations of these studies include slow enrollment, selection bias, high crossover rate, and inclusion of patients with mild deficit. To improve enrollment and minimize risk of diluting the overall treatment effect, futile recanalization and re-occlusion, optimal inclusion/exclusion criteria, including enrollment within 24 h of last known well, NIHSS score ≥ 10, pc-ASPECTS ≥ 8, no large pontine infarct, and the use of rescue therapy for underlying atherosclerotic stenosis, should be considered for future clinical trials.

## Introduction

Acute basilar artery occlusion (BAO) results in ischemia in brainstem, occipital lobes, and part of the thalami or cerebellum. Without reperfusion therapy, the rate of mortality or severe disability was as high as 90% [1, 2]. With intravenous or intra-arterial thrombolysis, the rate of death or dependency decreased to 78% and 76%, respectively [2, 3]. Although successful endovascular thrombectomy (EVT) for acute BAO was reported almost 2 decades ago [4, 5], its efficacy remains unproven.

In 2015, 5 landmark randomized controlled trials (RCTs) demonstrated powerful efficacy of EVT in patients with acute ischemic stroke (AIS) from large vessel occlusion in the anterior circulation (AC) within 6–12 h of symptom onset [6–10]. In 2018, DAWN and DEFUSE-3 showed similar efficacy in patients with salvageable ischemic penumbra within 16–24 h after last known well [11, 12]. These studies also revealed that EVT during extended time window was not associated with higher risk of symptomatic intracranial hemorrhage (sICH). The aim of this review was to highlight the latest findings of EVT for acute BAO and critical thinking on future study designs.

## Methods

We searched the PubMed for acute basilar artery occlusion and thrombectomy from January 2000 to October 2021. All retrospective studies and prospective registries with sample size ≥ 100 and functional outcome data at 90 days in the peer-reviewed journals were included. If the authors published sequential papers with accumulating numbers of cases, only the most recent publication was included. Studies without 90 days or long-term functional outcome data were excluded. All published RCTs were also included.

Favorable outcome in retrospective studies or prospective registries was defined as modified Rankin Scale (mRS) score 0–2 at 90 days. Primary outcome in the recently published RCTs was defined as mRS 0–3 at 90 days. sICH and mortality were compared as safety outcome.

## Findings and Discussion

### Retrospective Studies

Due to poor natural history, significant numbers of patients with acute BAO were treated with EVT empirically worldwide in the last 2 decades. Eighteen published retrospective studies were found to have sample size ≥ 100 and functional outcome data at 90 days [13–30]. In these studies, most patients had median or mean National Institutes of Health Stroke Scale (NIHSS) scores 14–31. The rate of receiving intravenous thrombolysis (IVT) varied from 14 to 45.1%. The mean or median onset to treatment time ranged from 3.8 to 10.4 h. Stent retrievers (SR) and/or direct aspiration (DA) device were used in most studies. Some studies also used rescue therapy (RT) with intra-arterial tissue plasminogen activator (tPA), angioplasty, or stenting.

The rate of favorable outcome (mRS score 0–2) at 90 days ranged from 27.1 to 46.9%.

Multivariate logistic regression analysis revealed that time to recanalization, early neurological improvement, good collateral circulation, and distal BAO were independent predictors of favorable outcome after EVT [17, 22, 24]. In contrast, older age, higher baseline NIHSS score, pc-ASPECTS < 8, extensive baseline infarction, large pontine infarct (infarct core ≥ 70% of pons), and sICH were independent predictors of poor outcome [14, 18, 25].

Analysis of the pooled data from the 18 studies showed 36.4% favorable outcome at 90 days and 4.6% sICH (Table 1). In comparison, the meta-analysis of the pooled data from the 5 landmark RCTs in the AC showed 46% favorable outcome at 90 days and 4% sICH [31]. A recent meta-analysis of EVT for patients with posterior circulation (PC) stroke showed a higher rate of futile recanalization, poorer outcome, but lower rate of sICH than the AC stroke [32].

**Table 1:** Retrospective studies of endovascular thrombectomy for acute basilar artery occlusion

Studies	Cases (*n*)	NIHSS scores	IVT (%)	Median or mean OTT (h)	EVT device	Favorable outcome (%)	sICH (%)	Mortality (%)
Mokin et al. [13]	100	19.2 ± 8.2	32	9.4 ± 7.8	SR, DA, RT	35	5	30
Bouslama et al. [14]	214	21 (12–28)	21	10.4 (4.8–18.7)	SR, DA, RT	27.1	–	46.7
Gory et al. [15]	117	–	–	–	SR, DA	36.5	1.7	43.9
Rentzos et al. [16]	110	31 (13–31)	14	5.0 (2.9–7.7)	IAT, SR, DA	35	9	41
Giorgianni et al. [17]	102	17	45.1	4.9	SR, DA, RT	38.2	4.9	30.5
Meinel et al. [18]	165	18 (8–30)	43.0	5.0 (3.5–8.0)	IAT, SR, RT	36.2	4.8	36.2
Lee et al. [19]	194	–	26.8	–	SR, RT	44.8	–	–
Kang et al. [20]	212	17 (10–24)	30.7	4 (2.8–6.0)	SR, DA, RT	44.8	1.9	16
Weber et al. [21]	139	12 (6–21)	41.7	3.8 (2.7–7.1)	SR, RT	38.0	0	33.7
Ravindren et al. [22]	231	14	–	–	SR, DA	29.5	6.0	36.8
Sun et al. [23]	187	22 (10–34)	19.3	7 (5–10)	SR, DA	36.4	4.3	20.3
Guenego et al. [24]	237	16 (9–39)	39	6.3 (3.8–7.6)	SR, DA, RT	38	–	39
Yoon et al. [25]	113	12 (7–19)	23.9	4.5 (3.8–6.0)	–	46.9	2.7	13.3
Wu et al. [26]	167	23 (15–33)	20.4	9.3 (5.6–13.0)	SR, DA, RT	30.5	6	31.7
Baik et al. [27]	161	17 (8–25)	25.5	4.7 (1.7–8.0)	SR, DA, RT	34.4	8.1	17.4
Ma et al. [28]	108	22 (12–34)	19.4	7 (5–10)	SR, RT	33.3	4.6	17.6
Sefcik et al. [29]	107	18.7 ± 9.3	18.2	–	DA, SR, RT	36.7	–	39.8
Alexandre et al. [30]	191	17 (7–20)	30	4.8 (3.4–7.0)	DA, SR	38.2	–	–
Pooled data	2514					36.4	4.6	30.0

Abbreviations: *DA*, direct aspiration; *EVT*, endovascular thrombectomy; *IVT*, intravenous thrombolysis; *NIHSS*, National Institutes of Health Stroke Scale; *OTT*, onset to treatment time; *RT*, rescue therapy; *sICH*, symptomatic intracranial hemorrhage; *SR*, stent retriever

Favorable outcome was defined as modified Rankin Scale score 0–2 at 90 days

Two large cohort studies compared functional outcome and safety of EVT for large vessel occlusion in the PC vs. AC (Table 2) [18, 21]. Meinel et al. examined the outcome of EVT in the multicenter BEYOND-SWIFT registry [18]. There were 165 patients with acute BAO in the PC and 1574 patients with large vessel occlusion in the AC. When adjusting for baseline characteristics, there was no significant difference in favorable outcome between the 2 groups (36.2% vs. 42.9%, adjusted odds ratio [OR] 0.986; 95% confidence interval [CI] 0.553–1.758, *p* = 0.12). However, BAO was associated with increased rate of futile recanalization (adjusted OR 2.146; 95% CI 1.267–3.633). Predictors for futile recanalization were older age, higher stroke severity, higher maneuver count, and intracranial stenting. There was no significant difference in sICH (4.8% vs. 6.3%, *p* = 0.608) between the 2 groups.

**Table 2:** Outcome of thrombectomy for large vessel occlusion in the anterior vs. posterior circulation

Studies		Cases (*n*)	Median NIHSS scores	IVT (%)	OTT (h)	Favorable Outcome (%)	sICH (%)	Mortality (%)
Meinel et al. [18]	BAO	165	18 (8–30)	43.0	5 (3.5–6)	36.2	4.8	36.2
	AC	1574	17 (12–20) *p* = 0.046	49.5 *p* = 0.12	3.8 (2.8–5.3) *p* < 0.001	42.9 *p* = 0.12	6.3 *p* = 0.608	24.4 *p* = 0.002
Weber et al. [21]	PC	139	12 (6–21)	41.7	3.8 (2.7–7.1)	38.0	0	33.7
	AC	961	15 (12–19) *p* = 0.024	52.5 *p* = 0.016	3.3 (2.3–4.7) *p* = 0.001	42.6 *p* = 0.392	3 *p* = 0.010	30.8 *p* = 0.539

Abbreviations: *AC*, anterior circulation; *BAO*, basilar artery occlusion; *IVT*, intravenous thrombolysis; *OTT*, onset to treatment time; *PC*, posterior circulation; *sICH*, symptomatic intracranial hemorrhage

Favorable outcome was defined as mRS 0–2 at 3 months

Weber et al. conducted a prospective multicenter registry on Revascularization in Ischemic Stroke Patients (REVASK) in Germany [21]. They compared the demographics, periprocedural times, complications, recanalization rates, and functional outcome between 139 consecutive patients with PC stroke (84.9% BAO) and 961 patients with AC stroke. Despite the significantly lower rate of IVT and delay in onset to treatment time in the PC group, there was no significant difference in favorable outcome (38.0% vs. 42.6%, *p* = 0.392) or mortality (33.7% vs. 30.8%, *p* = 0.539) at 90 days between the 2 groups.

These data indicated comparable efficacy and safety profile of EVT in the PC and AC.

### Prospective Studies

Table 3 shows the data from 2 large prospective studies of EVT for acute BAO [33, 34]. In 2015, Singer et al. reported the results of an investigator-initiated multicenter registry of EVT for acute BAO [33]. In 148 consecutive patients with BAO, 59% received IVT prior to EVT and 34% had favorable outcome at 90 days. Initial stroke severity and the collateral status predict clinical outcome.

**Table 3:** Results from prospective registries of EVT for acute basilar artery occlusion

Studies	Cases (*n*)	Median NIHSS scores	IVT (%)	Median OTT (h)	Favorable outcome (%)	sICH (%)	Mortality (%)
Singer et al. [33]	148	20	59	–	34	5.4	35
Zi et al. [34]							
EVT	647	27 (17–33)	18.4	4.0 (2.2–6.5)	27.4	7.1	46.2
Medical treatment	182	26.5 (16–33)	25.8	3.7 (1.9–6.8)	7.1 *p* ≤ 0.01	0.5 *p* < 0.01	71.4 *p* < 0.01

Abbreviations: *EVT*, endovascular thrombectomy; *IVT*, intravenous thrombolysis; *OTT*, onset to treatment time; *NIHSS*, National Institutes of Health Stroke Scale

Favorable outcome was defined as mRS score 0–2 at 90 days

The EVT for acute BAO (BASILAR) study was a non-randomized prospective registry of consecutive patients at 47 comprehensive stroke centers in China between January 2014 and May 2019 [34]. Patients with acute BAO within 24 h of last known well were enrolled. Among the 829 participants, 647 were treated with EVT plus standard medical therapy and 182 were treated with standard medical treatment alone. There was no difference in median NIHSS score between the 2 groups (27 vs. 26.5). The rate of favorable outcome (mRS 0–2) at 90 days was significantly higher in the EVT group than in the medical group (27.4% vs. 7.1%; *p* < 0.001). Moreover, EVT was associated with a significantly lower rate of 90-day mortality (adjusted OR 2.93; 95% CI 1.95–4.40; *p* < 0.001) despite the higher rate of sICH (7.1% vs. 0.5%; *p* < 0.001). The number needed to treat was 4.4 for 1 additional patient to be able to walk unassisted.

In a subsequent analysis of the impact of baseline posterior circulation Acute Stroke Prognosis Early Computed Tomography Score (pc-ASPECTS) on the efficacy of EVT for acute BAO, patients with pc-ASPECTS ≥ 8 could significantly benefit from EVT [35].

### Randomized Controlled Trials

Endovascular treatment vs. standard medical treatment for vertebrobasilar artery occlusion (BEST) is a multicenter open label RCT [36]. Between April 27, 2015 and September 27, 2017, 131 patients were randomly assigned (1:1) to EVT plus standard medical therapy or standard medical therapy alone within 8 h of symptom onset at 28 centers in China. The trial was stopped prematurely per recommendation of the data and safety monitoring board due to high crossover rate and poor enrollment. Fourteen (22%) of the 65 patients in the control group crossed over to receive EVT. In the EVT group, 3 (5%) of the 66 patients were unable to get EVT.

In the intention-to-treat analysis (Table 4), there was no significant difference in both primary outcome (42% vs. 32%, *p* = 0.23; adjusted OR 1.74; 95% CI 0.81–3.74) and favorable outcome (33% vs. 28%; *p* = 0.43, adjusted OR 1.40; 95% CI 0.64–3.10) at 90 days. The 90-day mortality was similar between the 2 groups (33% vs. 38%; *p* = 0.54) despite a higher rate of sICH in the EVT group (8% vs. 0%, *p* = 0.06).

**Table 4:** Results of randomized controlled trials of EVT for acute basilar artery occlusion

		Cases (*n*)	Median NIHSS	IVT (%)	Mortality (%)	sICH (%)	Favorable outcome (%)	Primary outcome (%)	Adjusted OR (95% CI)
BEST trial [36]									
Intention-to-treat	EVT	66	32 (18–38)	27	33	8	33	42	
	Control	65	26 (13–37)	32	38 *p* = 0.54	0 *p* = 0.06	28 *p* = 0.48	32 *p* = 0.23	1.7 (0.8–3.7)
Per protocol	EVT	63					35	44	
	Control	51					20	25	2.9 (1.2–7.0)
As treated	EVT	77					39	47	
	Control	54					19	24	3.0 (1.3–7.0)
BASICS trial [37]									
	EVT	154	21	78.6	38.3	4.5	35.1	44.2	
	Control	146	22	79.5	43.2 *p* = 0.29	0.7 *p* = 0.06	30.1	37.7 *p* = 0.19	1.2 (0.9–1.5)

Abbreviations: *EVT*, endovascular thrombectomy; *IVT*, intravenous thrombolysis; *OR*, odds ratio; *OTT*, onset to treatment time; *NIHSS*, National Institutes of Health Stroke Scale

Primary outcome was defined as mRS score 0–3 at 90 days

Favorable outcome was defined as mRS score 0–2 at 90 days

In the secondary analyses to assess the effect of crossovers, there was significantly higher rate of primary outcome in patients who received EVT in both per-protocol (44% vs. 25%; adjusted OR 2.90, 95% CI 1.20–7.03) and as-treated analysis (47% vs. 24%; adjusted OR 3.02; 95% CI 1.31–7.00). There was also a higher rate of favorable outcome and significant improvement in the overall distribution of 90-day mRS scores in the EVT group.

The Basilar Artery International Cooperation Study (BASICS) was conducted at 23 centers in 7 countries [37]. Between October 2011 and December 2019, 300 patients were randomized to EVT plus standard medical therapy or standard medical therapy alone within 6 h of estimated BAO. The study showed no significant differences in primary outcome (mRS score 0–3) at 90 days (44.2% vs. 37.7%, OR 1.18, 95% CI 0.92 to 1.50), favorable outcome (mRS score 0–2) at 90 days (35.1% and 30.1%), or sICH (4.5% vs. 0.7, OR 6.9; 95% CI 0.92 to 53.0) between the 2 groups. In subgroup analysis, there was a significant signal favoring EVT in patients with moderate to severe stroke (NIHSS ≥ 10) or pc-ASPECTS ≥ 8.

Of note, BASICS trial only enrolled 91 patients in the first 4 years. The inclusion criteria were then modified to allow enrollment of patients with age ≥ 85 and NIHSS score < 10. Patients with a mild deficit (NIHSS score < 10) in the medical group were shown to have a higher rate of primary outcome at 90 days than in the EVT group (80% vs. 65%), diluting the overall treatment effect of EVT.

In addition, 55% of screened patients in the BEST trial and 29.2% eligible patients in the BASIC trial were not enrolled for the studies, likely introducing significant selection bias [36–38].

### Meta-analysis

In a recent meta-analysis of five studies (2 RCTs and 3 observational cohorts) including a total of 1098 patients [39], patients receiving EVT had a nonsignificant trend towards mRS score 0–2 (RR 1.02, 95% CI 0.74–1.41), mRS score 0–3 (RR = 0.97, 95% CI 0.64–1.47), and overall functional improvement (OR 0.93, 95% CI 0.57–1.51) at 90 days.

In an aggregated meta-analysis of the BEST and BASICS trials with a Bayesian approach, EVT was associated with favorable outcomes (OR 1.62; 95% CI 1.01–2.77) and the number needed to treat would be 13 [40].

### Current Practice Guidelines

The American Heart Association and American Stroke Association guidelines published in 2018 stated that there is uncertainty about the benefit of EVT in acute BAO. Thrombectomy may be reasonable only in carefully selected patients within 6 h of stroke onset (class IIb; level of evidence C) [41].

## Critical Thinking on Future Study Design

The main limitations of the BEST and BASICS trials are poor enrollment, high crossover rate, inclusion of patients with mild deficit (NIHSS score < 10), and significant selection bias due to high proportion of eligible patients being treated outside the trials (55% of screened patients in the BEST and 29% in the BASICS) [36–38]. It is therefore essential to explore potential opportunities for improvement in future study design for EVT in acute BAO.

### Time Window for Future RCTs

The BEST and BASICS trials enrolled patients within 6 or 8 h of estimated BAO [36, 37]. This may partly explain the poor enrollment of the 2 studies. BASILAR study enrolled 829 patients within 24 h of last known well and demonstrated significant benefit of EVT [34]. In the ENDOSTROKE study, no significant association was found between onset to treatment time and clinical outcome [33]. In the absence of extensive baseline infarction or large pontine infarct, recanalization of BAO up to 48 h after symptom onset was found often safe and potentially effective [27, 28, 30, 42–44]. EVT in extended time windows was not associated with increased risk of sICH [42, 45].

Based on these findings, we propose to enroll patients within 24 h of last know well in future RCTs of EVT for acute BAO.

### Excluding Patients with Mild Deficit

Previous observational study demonstrated that patients with mild deficit (NIHSS score < 10) would not benefit from EVT [2]. In addition, the BASICS trial showed that patients with mild deficit (NIHSS score < 10) in the medical therapy group had a higher rate of primary outcome at 90 days than in the EVT group (80% vs. 65%) [37]. Therefore, patients with mild deficit may dilute the treatment effect of EVT and should be excluded for future RCTs.

### Futile Recanalization

Futile recanalization was defined as a technical success without meaningful improvement in functional outcome [18, 25, 26]. Compared with large vessel occlusion in the AC, BAO was associated with increased rate of futile recanalization [18, 32]. Extensive baseline infarction or large pontine infarct was found to be associated with higher rate of futile recanalization and poor outcome at 90 days. [18, 25, 26, 30, 33] The BASICS trial showed higher basilar artery patency at 24 h post-enrollment in the EVT group than in medical group (84.5% vs. 56.3%) [37], suggesting a significant rate of futile recanalization.

Therefore, patients with extensive baseline infarction (pc-ASPECTS < 8) or large pontine infarct (defined as infarct core ≥ 70% of the entire pons) should be excluded in future RCTs to reduce the rate of futile recanalization [25, 42].

### Imaging Strategy for Screening Eligible Patients

**Fig. 1:**
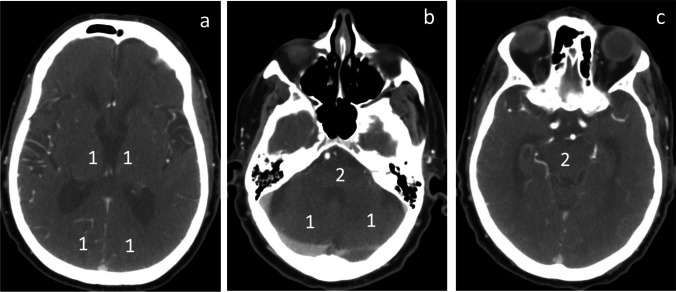
Posterior circulation Acute Stroke Prognosis Early CT Score (pc-ASPECTS) on CTA source image (CTASI). From 10 points, 1 point each (as indicated) is subtracted for hypoattenuation in the left or right thalamus, posterior cerebral artery territory (**a**), or cerebellar hemisphere (**b**), respectively, and 2 points each for hypoattenuation in any part of pons (**b**) or midbrain (**c**). pc-ASPECTS = 10 indicates a normal scan

Currently, there is no substantial data to support specific imaging strategy for screening patients for EVT in the PC [46]. The DAWN trial demonstrated significant benefit of EVT in patients with a mismatch between the severity of clinical deficit and infarct volume within 24 h of last known well [12]. PC-ASPECTS on CTA source image (CTASI) was developed to quantify the extent of early ischemic changes in the posterior circulation with a 10-point grading system (Fig. 1) [47]. From 10 points, 1 point each is subtracted for hypoattenuation on CTASI in the left or right thalamus, posterior cerebral artery territory, or cerebellar hemisphere, respectively, and 2 points each for hypoattenuation in any part of the pons or midbrain [47]. A pc-ASPECTS score of 10 indicates absence of visible posterior circulation ischemia. A score of 0 indicates visible ischemic changes in all pc-ASPECTS territories.

Compared with non-contrast CT images, pc-ASPECTS score on CTASI was shown to improve the overall sensitivity to detect ischemic changes in the posterior circulation [48, 49]. The extent of hypoattenuation on CTASI was significantly correlated with the final infarctions in patients with BAO [49]. The areas of the pontine infarct core and whole pons can also be measured on CTASI that displays the largest hypodense lesion in the pons [25, 49]. The pontine infarct core size was expressed as the percentage of infarct core area relative to the whole pontine area on the selected slice. Pontine infarct core ≥ 70% of the pons is considered large pontine infarct [25].

Of note, there was no difference in outcome detected in relation to the pc-ASPECTS in the subgroup analysis of BASICS [37]. This might be partly due to the measurement of pc-ASPECTS on non-contrast CT images in 54% of study patients (129 in the endovascular therapy group and 115 in the medical therapy group) and enrollment of significant numbers of patients with a mild deficit (NIHSS score < 10). Patients with mild deficit had higher rate of favorable outcome at 90 days in the medical treatment group. Inclusion of these patients in the BASICS trial likely diluted the treatment effect of EVT [37]. Therefore, we recommend CTASI for the measurement of pc-ASPECTS and exclusion of patients with NIHSS score < 10 in future RCTs.

MRI DWI provides better sensitivity for detection of early ischemic changes but may cause delay in door to puncture time for EVT [46]. Numerous studies demonstrated that pc-ASPECTS ≥ 8 was associated with favorable functional outcome after EVT [22, 25, 35, 49, 50]. In a cost-effectiveness analysis of initial imaging selection, comprehensive CT (non-contrast CT + CTA + CTP) at the time of presentation was shown to be the most cost-effective initial imaging strategy for EVT in AIS [51]. CTA/CTP was used for screening patients promptly for EVT in patients with BAO [22, 35, 49, 50].

A recently published retrospective study showed that perfusion imaging may predict favorable outcomes after basilar artery thrombectomy [52]. In a cohort of 103 patients, a Critical Area Perfusion Score (CAPS) of 0–6 points was used to quantify severe hypoperfusion (Tmax > 10) in cerebellum (1 point/hemisphere), pons (2 points), midbrain and/or thalamus (2 points). Patients were dichotomized into favorable (CAPS ≤ 3) and unfavorable (CAPS > 3) groups. Patient with CAPS ≤ 3 (87%) had a lower median NIHSS score (12.5, IQR 7–22) compared to patients with CAPS > 3 (23, IQR 19–36; *p* = 0.01). Reperfusion was achieved in 84% of all patients. Sixty-four percent of reperfused CAPS ≤ 3 patients had a favorable outcome compared to 8% of nonreperfused CAPS ≤ 3 patients (OR 21.0, 95% CI 2.6–170; *p* < 0.001). No CAPS > 3 patients had a favorable outcome, regardless of reperfusion. In a multivariate regression analysis, CAPS ≤ 3 was a robust independent predictor of favorable outcome after adjustment for reperfusion, age, and initial NIHSS score (OR 39.25, 95% CI 1.34– > 999, *p* = 0.04). Therefore, cerebral perfusion imaging profile may be considered to identify eligible patients for future RCTs.

Another recent study compared patients with BAO and anterior circulation LVO using propensity score matching [53]. Multivariate logistic regression analysis did not show a significant difference in functional outcome between BAO and anterior circulation LVO. However, in patients with an onset-to-door-time ≥ 270 min, EVT of BAO was associated with poor functional outcome as compared to anterior circulation LVO.

Therefore, cerebral perfusion imaging might be a useful tool for selection of eligible patients in future studies, especially in those arriving late [52, 53].

**Fig. 2:**
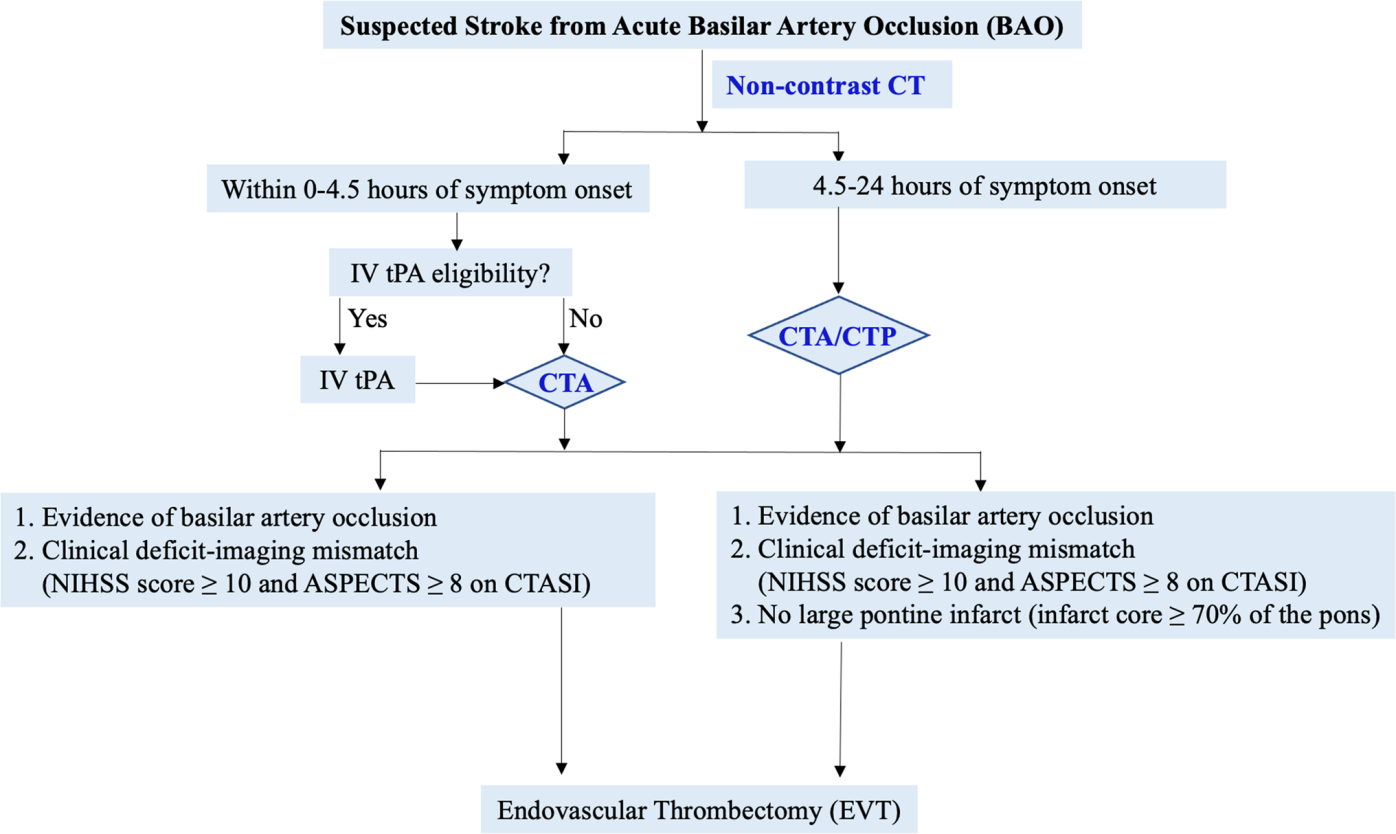
Simple imaging guide for screening patients with acute BAO for EVT

Here, we propose a simple imaging guide for screening patients with suspected acute BAO for EVT (Fig. 2). Non-contrast CT is performed to evaluate tPA eligibility. CTA/CTP is then performed to evaluate BAO, pc-ASPECTS score, and infarct volume. Patient is eligible for enrollment in RCTs if there is a significant mismatch between the severity of clinical deficit and infarct volume (NIHSS score ≥ 10 and ASPECTS ≥ 8) [37, 46, 48]. Patients with large pontine infarct (infarct core ≥ 70% of the pons) should be excluded to minimize futile recanalization. [25].

### Rescue Therapy

Approximately 24–47% of patients with acute BAO were found to have underlying intracranial atherosclerotic disease and superimposed in situ thrombosis [19, 26, 29, 54–57]. Patients with underlying intracranial stenosis were found to have longer procedure time, lower recanalization rate, or higher rate of re-occlusion than those with embolic occlusion [19, 26, 54, 55]. They were also found to have lower rate of favorable outcome than patients with embolic occlusion (37.9% vs. 62.1%, 20% vs. 53%, or 10.5% vs. 37.5, respectively) [19, 54, 56].

Intraprocedural use of glycoprotein IIb/IIIa inhibitor, percutaneous transluminal angioplasty, or stenting for underlying intracranial stenosis as rescue therapy was shown to improve the rate of functional outcome. With the use of rescue therapy, there was no significant difference in the rates of favorable outcome between in situ atherosclerotic thrombosis group and embolic group (26.9% vs. 33.7%, 37.5% vs. 41.5%, or 60% vs. 51%, respectively) [26, 55, 57].

Recently, Luo et al. reported prospective registry data on rescue therapy for acute BAO [58]. In a cohort of 93 patients who failed EVT, 81 (87.1%) received rescue therapy with a 92.6% recanalization rate. Compared with patients who did not receive rescue therapy (*n* = 12), the rescue therapy group had a significantly higher rate of favorable outcomes at 90 days (51.9% vs. 16.7%, *p* = 0.023). There was no significant increase in sICH.

Therefore, rescue therapy should be considered in patients with underlying atherosclerotic stenosis to improve outcome after EVT [26, 55, 57, 58].

### Primary Outcome Measure

All the landmark RCTs for EVT in the anterior circulation used mRS 0–2 as the primary outcome measure [6–12]. In contrast, the 2 RCTs of EVT for acute BAO used mRS 0–3 (moderate disability) as primary outcome measure [36, 37]. To be consistent with reporting standard, we suggest using mRS 0–2 as primary outcome measure in future studies. Of note, mRS 0–3 (moderate disability) represents reasonably good quality of life and may be used as secondary outcome.

## Conclusion

The efficacy of EVT for acute BAO in the posterior circulation remains unproven. Additional RCTs with optimal inclusion and exclusion criteria, e.g., enrollment within 24 h of last known well, NIHSS score ≥ 10, pc-ASPECTS ≥ 8, no large pontine infarct, and the use of rescue therapy for underlying atherosclerotic stenosis, are warranted.

## Abbreviations

AC: Anterior circulation
AIS: Acute ischemic stroke
ASPECTS: Alberta Stroke Program Early CT Score
CT: Computed tomography
EVT: Endovascular thrombectomy
IVT: Intravenous thrombolysis
OTT: Onset to treatment time
PC: Posterior circulation
RCTs: Randomized controlled trials
sICH: Symptomatic intracranial hemorrhage
